# Yoga for Adults with Type 2 Diabetes: A Systematic Review of Controlled Trials

**DOI:** 10.1155/2016/6979370

**Published:** 2015-12-14

**Authors:** Kim E. Innes, Terry Kit Selfe

**Affiliations:** ^1^Department of Epidemiology, West Virginia University School of Public Health, Morgantown, WV 26506, USA; ^2^Center for the Study of Complementary and Alternative Therapies, University of Virginia Health System, Charlottesville, VA 22903, USA

## Abstract

A growing body of evidence suggests yogic practices may benefit adults with type 2 diabetes (DM2). In this systematic review, we evaluate available evidence from prospective controlled trials regarding the effects of yoga-based programs on specific health outcomes pertinent to DM2 management. To identify qualifying studies, we searched nine databases and scanned bibliographies of relevant review papers and all identified articles. Controlled trials that did not target adults with diabetes, included only adults with type 1 diabetes, were under two-week duration, or did not include quantitative outcome data were excluded. Study quality was evaluated using the PEDro scale. Thirty-three papers reporting findings from 25 controlled trials (13 nonrandomized, 12 randomized) met our inclusion criteria (*N* = 2170 participants). Collectively, findings suggest that yogic practices may promote significant improvements in several indices of importance in DM2 management, including glycemic control, lipid levels, and body composition. More limited data suggest that yoga may also lower oxidative stress and blood pressure; enhance pulmonary and autonomic function, mood, sleep, and quality of life; and reduce medication use in adults with DM2. However, given the methodological limitations of existing studies, additional high-quality investigations are required to confirm and further elucidate the potential benefits of yoga programs in populations with DM2.

## 1. Introduction

Type 2 diabetes (DM2) has become a leading public health issue globally, with estimated 366 million people affected in 2011 [1]. This figure represents a more than twofold rise in the last three decades and parallels the growing pandemic of obesity and the increasingly widespread adoption of Western lifestyles [1, 2]. Worldwide prevalence is expected to continue rising in both industrialized and developing countries [2, 3], with numbers projected to reach 552 million adults by 2030 [1, 4]. DM2 is now a leading cause of death and disability and significantly increases risk for both macrovascular complications, such as atherosclerosis, and microvascular complications, such as retinitis, diabetic neuropathy, and renal disease [5]. DM2 is also strongly associated with elevated risk for other serious chronic conditions, including depression and dementia [3, 6]. Cardiovascular disease (CVD) is the primary cause of morbidity and mortality in those with DM2 [7], accounting for at least 65% of deaths in this population [8]. In addition, the social and economic burden of DM2 is substantial and growing rapidly. For example, DM2 accounts for at least 10 percent of all healthcare expenses in the United States, making DM2 the single most costly chronic disease [9].

DM2 is typified by hyperglycemia in the presence of insulin resistance [6]. Other key related hemodynamic and metabolic abnormalities characterizing DM2 include elevated blood pressure, dyslipidemia, and chronic inflammation, as well as hypercoagulation and increased oxidative stress [5, 10–12]. Risk for DM2 rises with increasing age and is further elevated in certain racial and ethnic groups, including non-Hispanic blacks, Asians, Native Americans, and Pacific Islanders [13, 14]. However, while race, age, genetic predisposition, and other nonmodifiable factors are important in the pathogenesis of DM2, lifestyle factors, particularly, physical inactivity, overnutrition, and related obesity, are thought to be primarily responsible for the current global diabetes epidemic [1, 15]. Other contributing lifestyle-related factors include chronic stress, impaired sleep, and smoking [2, 10, 16, 17]. In fact, lifestyle factors may account for 90% of incident diabetes cases [2] and are significant predictors of DM2-related complications and mortality [18]. Thus, a central element of DM2 care is lifestyle management, which is considered critical to the prevention of acute complications and the reduction of risk for long term complications [6].

A central goal in DM2 management is the reduction of blood glucose levels, which has been shown to reduce risk of microvascular and possibly certain other complications [3, 5, 6]. However, while improving glycemic control remains a priority, the critical importance of multifactorial DM2 management has been increasingly emphasized in recent years, reflecting the complex constellation of factors that underlie the development of DM2 and its sequelae [3, 6]. In particular, reducing CVD risk factors is crucial to effective DM management [6]. Additional goals include reducing DM-related distress, alleviating depression, and enhancing emotional well-being and quality of life, factors that are important predictors of glycemic control, complication rates, treatment adherence, and other outcomes [6, 19–24].

In light of the above, identifying sustainable lifestyle interventions with the potential to improve multiple factors of relevance to the management of this complex illness is of clear importance. Mind-body practices such as yoga, which capitalize on the ability of the mind to enhance physical health (and vice versa), appear particularly suited for addressing multifactorial conditions. Yoga is a traditional mind-body system originating in India over 4000 years ago [25]. In recent decades, the practice of yoga has been rising in both developed and developing countries worldwide [26–29], and the field of yoga therapy is now growing rapidly [30]. The goals of yoga, a Sanskrit term meaning “yoke or union,” do not center primarily on physical fitness, but rather on integration of mind, body, and spirit, cultivation of balance, calm, harmony, and awareness, and, in classic yoga traditions, the attainment of selflessness and spiritual enlightenment [25, 26]. Of the several major branches of yoga, the most widely practiced forms include Raja (royal or classical) yoga and the closely related Hatha yoga, sometimes known as the yoga of activity [25, 26]. Mantra yoga, emphasizing the use of specific sounds or chants to achieve mental and spiritual transformation, was popularized in the West by Maharishi Mahesh Yogi, the founder of Transcendental Meditation (TM). Hatha and Raja yoga emphasize specific postures (asanas), including both active and relaxation or restorative poses, as well as breath control (pranayama), concentration (dharana), and meditation (dhyana), with some schools focusing primarily on restorative/meditative practices. Hatha yoga, the branch of yoga most widely practiced in the US and other Western countries, also incorporates cleansing exercises, mantras or chants, and specific hand gestures (mudras). Originally developed to prepare the body for meditation, Hatha yoga itself encompasses many different styles, including Iyengar, Ashtanga, Integral, Kundalini, Viniyoga, Vinyasa, Kripalu, and Bikram yoga [25, 26].

A growing body of evidence suggests yoga practice may reduce risk for CVD and lead to improvements in physical health and well-being in a range of populations [31–37], potentially including those with DM2. In this systematic review, we critically evaluate available evidence from controlled trials regarding the effects of yoga-based programs on health-related outcomes in adults with DM2. We also outline major limitations in the current literature, briefly discuss possible mechanisms that may underlie observed benefits, and suggest directions for future research.

## 2. Methods

We searched eight databases via EbscoHost (Academic Search Complete, Alt HealthWatch, CINAHL with Full Text, Health Source: Nursing/Academic Edition, MEDLINE, PsycARTICLES, PsycINFO, and SPORTDiscus with Full Text) from their inceptions through March 2015 for original, scholarly articles reporting on controlled trials of yoga in the management of diabetes. The basic search strategy, (yoga OR yogic) AND diabetes, yielded 468 results; the same search conducted in the Cochrane Central Register of Controlled Trials resulted in 37 trials. A secondary search of the Physiotherapy Evidence Database (PEDro) for the terms yog^*∗*^ AND diabetes yielded 34 articles. Titles and abstracts of the citations were scanned to identify potential articles for inclusion in this review. Potentially eligible papers were retrieved for more detailed review. In addition, we manually searched our own files, the citation sections of all identified articles, and the reference sections of recent review articles regarding diabetes and yoga.

Original, controlled studies were included if they evaluated the effects of yoga and yoga-based interventions on measures of glycemic control and insulin resistance, lipid profiles, body weight or composition, blood pressure, or other relevant outcomes, such as oxidative stress, nervous system function, cardiopulmonary function, mood and sleep impairment, or medication use. We excluded uncontrolled trials, cross-sectional studies, case series, and case studies, as well as trials that were published only in dissertation or abstract form. We also excluded articles that did not specifically target adults with diabetes, involve an intervention focused on yoga as the major component, study a yoga program of at least two week's duration, or report quantitative outcome data. Studies targeting populations with type 1 diabetes were also excluded, as the etiology and management for type 1 diabetes differ from those for DM2.

We categorized clinical measures and outcomes evaluated into several domains. For each domain, we summarized findings from relevant studies. Study quality was evaluated using the PEDro scale, which scores papers based on the following 10 criteria: (1) subjects were randomly allocated to groups, (2) allocation was concealed, (3) the groups were similar at baseline regarding the most important prognostic indicators, (4) there was blinding of all subjects, (5) there was blinding of all therapists who administered the therapy, (6) there was blinding of all assessors who measured at least one key outcome, (7) measures of at least one key outcome were obtained from more than 85% of the subjects initially allocated to groups, (8) all subjects for whom outcome measures were available received the treatment or control condition as allocated or, where this was not the case, data for at least one key outcome was analyzed by “intention to treat,” (9) the results of between group statistical comparisons are reported for at least one key outcome, and (10) the study provides both point measures and measures of variability for at least one key outcome. One point is awarded for each criterion reported in the paper. In addition, PEDro assesses whether study eligibility criteria were specified; however, as this does not address statistical or internal validity, no points are awarded for this criterion. Whenever available, we reported the score given in the PEDro database; when the study was not in PEDro, we assigned a score based on the PEDro instructional materials (http://www.pedro.org.au/wp-content/uploads/PEDro_scale.pdf). In our discussion of findings, we also considered recent meta-analyses of RCTs targeting populations with diabetes.

Each eligible study identified was classified into one of two design categories: randomized controlled trials (RCTs) or nonrandomized controlled trials (NRCTs). In RCTs, each participant is randomly assigned to one of at least two groups: an active intervention (i.e., yoga) group and one or more comparator groups. In NRCTs, the experimental design and analytic strategy are similar to that of RCT's, but the allocation to a given treatment is not performed randomly. Articles were selected and data extracted and evaluated by both authors; conflicts were resolved by discussion and consensus.

## 3. Results 

Of 539 potentially relevant abstracts and citation indices scanned, 159 potentially eligible papers were identified for closer review. A total of 33 papers reporting findings from 25 original studies, including 13 nonrandomized controlled trials (NRCTs) [38–50] and 12 randomized controlled trials (RCTs) [51–70], representing a total of 2170 enrolled participants met our eligibility criteria and were included in this review.

Of the 25 studies, 24 (96%) specified adults with DM2; in the one remaining investigation, participants were recruited from a diabetes clinic, but authors did not specify type of diabetes (Table 1). Age range was wide in most studies, with some specifying no upper age limit [57, 60–62, 65]. Of the 23 studies that specified age cut-offs (12 NRCT, 11 RCTs), 8 (34.8%) excluded patients under 30/35 years of age; an additional 10 (43.5%) excluded those under 40/45 years of age. While several studies included adults over the age of 65 [61, 62, 66–70], only one study specifically targeted elder adults [61, 62]. All but two studies [51, 52, 60] included participants of both genders; likewise, only two studies clearly specified exclusion of patients on anti-DM medications [38, 43]. Patients ranged from newly or recently diagnosed DM2 to those diagnosed with DM2 for 10 years or more, often within the same study, with stated exclusion and inclusion criteria varying from study to study. Of the 17 studies with sufficient detail to allow determination, all but 2 [45, 65] excluded patients with significant complications (Table 1), although, again, exclusion conditions were not uniform across studies.

All but 2 were published in 2000 or later, with the majority (56%) being published in the last 5 years. Most (80%) of the studies were conducted in India. The vast majority, 76%, were moderate-sized studies that included over 40 participants, with 44% including 61 or more participants and 8 (32%) including at least 100 participants. Yoga interventions ranged from 15 days [60] to 12 months [69] in duration, with 72% including at least 12 weeks of practice (Table 1). Programs varied substantially in practice frequency, intensity, and content, including, for example, a 3-month Hatha yoga program in which participants attended 1-2 classes/week [65], a 90-day program of daily deep yoga relaxation practice (yoga nidra) [64], a 6-month Sudarshan Kriya (SKY) rhythmic breathing program, with classes once/week and daily home practice [56], and 3- to 12-month comprehensive yoga program with practice 6 to 7 days/week [57]. Ninety-two percent of the yoga programs incorporated active asanas or yoga poses. Comparator conditions also varied widely, from wait list/no treatment [43, 65], standard care [39–42, 45–51, 54–56, 58, 63–66, 70], and/or group education [61] to a comprehensive conventional exercise program [57, 60, 66, 69]. Three studies included more than one comparator group [38, 63, 66]. For some studies, standard care included only medications [39, 40, 48, 49, 58, 63, 64], whereas in others, standard care also included a special diet [38, 41, 42, 47, 50, 54, 56, 70] and/or walking or other exercises [47, 48, 50, 54–56].

### 3.1. Effects of Yoga on Metabolic, Anthropometric, and Hemodynamic Indices

Twenty-five eligible studies assessed the potential impact of yoga-based programs on one or more anthropometric or physiologic markers of importance to diabetes management and prognosis, including measures of glucose tolerance and insulin resistance, lipid profiles, body weight and composition, and blood pressure. Findings of these studies are summarized in Table 2 and are discussed briefly below.

#### 3.1.1. Measures of Glycemia and Insulin Resistance

Twenty-four studies investigated the effects of yoga on markers of glycemia and insulin resistance, with all but two documenting significant, postintervention improvement in one or more measures following the practice of yoga either alone or in combination with other therapies. All but two programs [55, 64] incorporated active yoga asanas or poses; interventions ranged in duration from 15 days [60] to 12 months [69]. Of the 12 NRCTs (total *N* = 1090 participants), all reported significant improvement in one or more indices of glycemia/insulin resistance; reductions in postprandial blood glucose (PPBG) [40, 42–46, 48], fasting blood glucose (FBG) [38–49], fasting insulin [48], fructosamine [38], and glycosylated hemoglobin (HbA1c) [39, 41–45, 49] were observed among participants completing a yoga-based intervention compared to controls receiving no treatment [43], standard care [39–42, 44–46, 48, 49], standard care with light exercise [47], or a low fat vegetarian diet alone [38].

Of the 12 RCTs evaluating the effects of yogic practices on indices of glycemia and insulin resistance (*N* = 1040 participants), 10 reported significant reductions in at least one measure (Tables 2 and 3). Reported improvements again included significant declines in PPBG [53, 55, 63, 64], FBG [51, 59–61, 63, 64, 66, 70], insulin [51], and HbA1c [61, 66, 70] in those assigned to a yoga-based program versus standard care [51, 55, 58, 63, 64, 66, 67, 70], group education [61], or brisk walking [60]. Two additional trials observed significant beneficial changes in PPBG, FBG, and HbA1c following a comprehensive yoga program [57, 66] that were similar to those detected in participants assigned to a relatively intensive exercise intervention [57, 66].

In contrast, two RCTs documented no significant changes in these parameters. In a small 12-month Cuban trial of 40 adults with uncomplicated DM2, yoga group participants showed modest declines in FBG (7%) that did not differ significantly from those of the exercise group (1.7%) [69]. Similarly, a British study of 59 participants with DM2 showed no significant improvements either in glucose control [65], findings that may be in part attributable to low rates of participant compliance (50% class attendance and 0% performance of home practice).

Collectively, 92% of the controlled trials reviewed, including 12/12 NRCTs and 10/12 RCTs, reported improvements in glucose control with yoga-based programs that were statistically and clinically significant, suggesting that yogic practices may improve glucose control in adults with DM2. However, between group comparisons were not reported in ten studies (7 NRCTs [38, 40, 43, 44, 46–48], 3 RCTs [60, 63, 64]), and, as discussed below, additional methodologic or other limitations plagued most trials, hindering interpretation of findings. Magnitude of effects varied substantially among studies, from relatively modest to very large improvements depending on the study design, population, and comparator group (Table 3).

#### 3.1.2. Lipid Profiles

Of the 25 studies included in this review, 16 (*N* = 1575 total participants) examined the effects of yoga programs on lipid profiles in those with DM2, with all but one trial [65] reporting significant improvement in one or more lipid indices (Tables 2–4). Studies all incorporated active yoga asanas and varied in length from 40 days [42] to 12 months [69]; in all except one trial [65], participants assigned to the yoga intervention practiced at least 3 days/week. As indicated in Table 2, all 8 NRCTs (*N* = 737 participants) reported significant improvements in lipid profiles, including reductions in levels of total cholesterol (TC), low-density lipoprotein cholesterol (LDL), very low-density lipoprotein cholesterol (VLDL), and triglycerides (Tg), and increases in high-density lipoprotein cholesterol (HDL) relative to standard care [38–40, 42, 44, 48, 49], or standard care with light exercise [47].

Likewise, 7 of the 8 RCTs (totaling 838 participants) reported significant beneficial changes in serum lipids following completion of a yoga-based program. These included significant declines in TC [58, 61, 63, 66, 69], LDL [57, 58, 61, 63, 69], and Tg [51, 58, 61, 69] and significant increases in HDL [57, 58, 61, 63, 66, 69] relative to standard care [51, 58, 63, 66], group education [61], or a moderate intensity exercise program [57, 69]. Only one RCT, a 12-week UK study of 59 adults, reported no significant improvement in any of the serum lipids following the yoga program [65], findings, again, likely in part attributable to poor participant compliance.

Overall, 94% of the studies reviewed reported improvements in lipid levels following completion of yogic programs of varying intensity and duration. Again, reported changes were clinically as well as statistically significant, with magnitude of effects varying substantially with the study and target population (Table 3). However, again, interpretation of findings is hampered by the lack of between group comparisons in several studies, as well as other design, methodologic, and/or reporting limitations characterizing many of the studies, discussed in more detail below.

#### 3.1.3. Body Weight and Composition

Nine controlled trials have evaluated the potential influence of yoga on body weight and composition in adults with DM2, including 6 NRCTs and 3 RCTs; again, all but one study [65] (89%) documented improvements in those assigned to a yoga intervention. All of the yoga programs included active asanas. Trials ranged from 40 days [47] to 6 months [66] in duration, and, in all but the one trial reporting no significant benefits [65], participants practiced at least 3 times per week. Of the 6 NRCTs (*N* = 613 total participants), all reported significant beneficial changes in at least one measure of body composition, including reductions in weight [38, 44, 48], body mass index (BMI) [44, 45, 48, 49], and waist-hip ratio [44, 47, 49] relative to standard care [38, 44, 45, 48, 49] or standard care and light exercise [47]. Likewise, 2 of the 3 RCTs (*N* = 390 total participants), including a 6-month Cuban study in 231 adults [66] and a 3-month Indian trial of 100 adults [58], reported significant decreases in weight [58], BMI [66], and waist-hip ratio [58] in participants assigned to a yoga program versus standard care; yoga group improvements were again comparable to those documented following a moderate intensity physical activity program [66]. In contrast, a UK study of 59 adults showed no significant reduction in weight or BMI relative to standard care [65], although poor participant adherence renders interpretation of findings problematic.

#### 3.1.4. Blood Pressure

Only 5 of the 25 controlled trials targeting adults with DM2 evaluated the potential effects of yogic practices on blood pressure, including 3 NRCTs and 2 RCTs (*N* = total of 588 participants). Trials varied in length from 40 days to 3 months, and all included active asanas. Three of the five studies, two NRCTs and one RCT [59], in Indian adults showed significant drops in systolic and diastolic blood pressure relative to standard care [49, 59] or standard care combined with walking [47]. Another Indian study of 123 adults [45] showed similar, but not statistically significant, declines in blood pressure in participants assigned to the yoga versus the standard care group. One exploratory RCT of British adults [65] did not present any quantitative data on blood pressure but reported no significant changes following a 12-week versus standard care program.

### 3.2. Observed Effects of Yoga on Other Pertinent Indices

There is mounting evidence, reviewed briefly below, that yoga may improve other risk indices pertinent to the DM2 management as well, including oxidative stress, impairments in mood and sleep, nervous system and pulmonary function, and medication usage.* Oxidative stress,* an imbalance between free radicals and antioxidants, has been strongly implicated in the development of DM2 and diabetes-related complications [74, 75]. For example, the oxidative imbalance that often characterizes DM2 can lead to prothrombotic changes, endothelial dysfunction, and chronic vascular inflammation, exacerbate insulin resistance and associated hyperglycemia, and ultimately promote organ damage; these reciprocally related changes are thought to mediate many of the atherosclerotic and thrombotic alterations associated with the metabolic syndrome and to play a central role in the pathogenesis and progression of diabetes and CVD [10, 75–78]. Thus, interventions that reduce oxidative stress in those with diabetes may aid in improving risk profiles and decreasing diabetes-related complications. To date, at least five controlled trials have examined the effects of yoga on measures of oxidative stress in adults with DM2. Yoga interventions all incorporated active asanas and ranged from 40 days to 6 months in duration; all indicated beneficial changes with yoga (Table 2). For example, four NRCTs in Indian adults documented improvements in indices of oxidative balance; these included significant declines in MDA [40, 45, 47] among participants completing a 40-day [47] to 4-month yoga program [40] versus those assigned to standard care [40, 45] or standard care with light exercise [47]; in their 3-month study of 123 adults, Hegde et al. also reported significantly greater increases in serum levels of the antioxidants glutathione and Vitamin C in the yoga versus standard care group [45]; likewise, Mahapure et al. noted significantly greater increases in superoxide dismutase (SOD) levels in the yoga group relative to the standard care control in their 6-week study [41]. Additionally, in an RCT of 231 Cuban adults, those assigned to a 24-week yoga program showed increases in activity of the antioxidant, SOD, and reductions in malondialdehyde (MDA), an estimate of lipid oxidative damage, that were comparable to those completing a 24-week conventional exercise program, and greater than participants assigned to standard care [66].

*Mood and sleep impairment,* common comorbid conditions in those with DM2, are reciprocally related to the development and progression of DM2 [16, 79]. For example, depression and sleep disturbance, both characterized by dysregulation of the hypothalamic-pituitary-adrenal (HPA) axis, sympathetic overactivity, and reduced cardiovagal tone [80–88], have been shown to increase subsequent risk for prediabetes [89] and incident diabetes [16, 90–98], impair metabolic control in persons with diabetes [99–104], and lead to excess morbidity and mortality [10, 22, 76, 93–95, 105–109]. However, although there is substantial evidence to suggest that yogic practices are helpful in reducing symptoms of depression and anxiety [35, 37, 80, 110, 111], alleviating stress [10, 80, 112, 113], improving sleep [80, 110, 111, 114–119], and enhancing psychological well-being [80, 112] in both healthy and clinical populations, few controlled trials have examined the effects of yoga on these endpoints in populations with DM2. Our search identified only four that specifically assessed indices of psychological status, including one NRCT [49] and three RCTs [53, 55, 64, 65]; significant improvements were noted with yogic practices in all but one study (Table 2). These included beneficial changes in multiple domains of quality of life [49, 53, 55], measures of psychological well-being [49], and symptoms of distress [64]. Studies regarding the effects of yoga on sleep in those with DM2 are even fewer. We identified only one trial of DM2 patients, an RCT conducted in India, that examined the effects of yoga on any measure of impaired sleep; in this study of 41 adults, participants completing a program of daily yoga nidra (a deep relaxation yoga practice) experienced a reduction in the prevalence of insomnia, from 43 to 5% [64].

#### 3.2.1. Nervous System Function

Autonomic nervous system dysfunction, a correlate of obesity and poor cardiorespiratory fitness, has been strongly and bidirectionally related to insulin resistance and hypertension, implicated in the development of diabetes [120, 121] and CVD [122], and associated with increased risk for morbidity and mortality in individuals with DM2 [123, 124]. While more than 30 controlled studies, including 17 RCTs, have evaluated the effect of yoga on markers of sympathetic/parasympathetic activation and cardiovagal function [125, 126], our search identified only three that targeted adults with DM2, all conducted in India. These include two NRCTs [47, 127] assessing the effects of 3-month program of simple pranayama exercises [127] and a 40-day comprehensive yoga intervention [47] and an RCT of SKY yoga (a cyclical breathing practice) in adults enrolled in a diabetes lifestyle modification program that included daily brisk walking [54, 55] (Table 2). Participants who completed a pranayama program showed significant improvements in multiple indices of cardiac autonomic function [47, 55] and significant reductions in heart [47] and respiratory rate [127], relative to controls receiving standard care [55, 127] or standard care combined with exercise [47]. These findings suggest that yogic practices may promote a reduction in sympathetic activation, enhancement of cardiovagal function, and a shift in autonomic nervous system balance from primarily sympathetic to parasympathetic in adults with DM2. In addition, findings of three recent NRCTs suggest that yoga may also help mitigate the central and peripheral nervous system damage associated with DM2. For example, following completion of a 45-day daily yoga program, participants showed significant declines in the latency of event-related potentials (ERP), a marker of higher brain, including cognitive and memory function, and increases in ERP amplitude compared to those assigned to standard care [46]. Likewise, adults with DM2 who completed a 40-day yoga intervention showed significant improvements in certain measures of median nerve conduction velocity and amplitude relative to controls [47, 50], although incomplete information and lack of between group comparisons limit interpretation of findings.

#### 3.2.2. Pulmonary Function

Compromised lung function is both an important complication [128, 129] and a significant predictor [130] of DM2 and has been inversely associated with insulin resistance [128] and glycemic exposure [129, 131]. While studies in adults with DM2 are limited, available data from two NRCTs support a possible beneficial influence of yogic practices on pulmonary function in this population [47, 127]. Reported improvements included significant increases in forced expiratory volume, forced vital capacity, peak expiratory flow rate, and maximum voluntary ventilation following completion of a 3-month program of simple yogic breathing exercises [127] or a 40-day comprehensive program [47].

#### 3.2.3. Medication Use

Three controlled trials to date (two NRCTs [44, 49], one RCT [57]) have shown significant reductions in diabetes medication use in patients completing a three- [44, 49] to nine-month [57] yoga program relative to those assigned to standard care [44, 49] or a comprehensive exercise program [57]. Some of these declines were quite substantial. For example, in their trial of 154 adults with diabetes, Agrawal et al. reported 26 to 40% reductions in medication use in the yoga group at the 3-month follow-up [49].

## 4. Discussion

Overall, findings of these studies suggest that yoga-based practices may have significant beneficial effects on multiple factors important in DM2 management and prevention, including glycemic control, insulin resistance, lipid profiles, body composition, and blood pressure. These findings are further supported by recently published meta-analyses regarding the effects of yoga on specific CVD risk factors of relevance to DM2, detailed in Table 4 [34, 71–73]. For example, in subanalyses restricted to RCTs in adults with DM2, Cramer et al. reported greater average declines of 26 mg/dL in FBG and 0.5% in HbA1c, greater mean reductions of 13 mg/dL in TC, 10 mg/dL in LDL, 5 mg/dL in VLDL, and 24 mg/dL in Tg levels, and a higher average increase of 6 mg/dL in HDL in participants assigned to a yoga versus standard care group [34]. Notably, the authors also found significantly greater reductions in LDL (9 mg/dL) and higher increases in HDL levels (4 mg/dL) in the yoga versus conventional exercise group. Likewise, Cramer et al. also reported greater mean reductions in waist-hip ratio in participants with DM2 assigned to a yoga versus a standard care group [34]. Similarly, in a subanalysis of RCTs in adults with diabetes or metabolic syndrome, Chu et al. reported a mean BMI reduction of over 1.6 kg/m^2^ in those in the yoga versus control group [71]. While studies regarding the effects of yoga on blood pressure in adults with DM2 remain few, recent meta-analyses regarding the effects of yoga on other populations at risk for CVD suggest that yoga may be beneficial for regulating blood pressure in patients with diabetes as well. For example, in subanalyses of RCTs limited to 8 trials of adults without frank diabetes but at high risk of DM2 and CVD (e.g., those with obesity, impaired glucose tolerance, or metabolic syndrome), the authors noted significant mean declines of 10 mmHg in systolic blood pressure and 7.5 mmHg in diastolic blood pressure in participants receiving a yoga intervention versus standard care [34]. Meta-analyses of controlled trials in adults with hypertension have yielded similar findings [72, 73], indicating significant declines of 8–10 mmHg in systolic blood pressure and 6-7 mmHg in diastolic blood pressure in the yoga versus standard care groups (Table 4).

More limited data suggest that yoga may also lower oxidative stress, decrease sympathetic activation and improve nervous system function, enhance pulmonary performance, mood, sleep, and quality of life, and reduce medication use in those with DM2. Relatively few studies have examined the effects of yogic practices on psychological status and sleep, which are frequently compromised in DM2 and significant contributors to diabetes progression. Likewise, studies evaluating the effects of yoga on clinical outcomes and certain emerging risk factors in DM2, including proinflammatory markers, remain sparse, and studies to determine optimal yoga dosing and program structure for different DM2 populations are lacking. As indicated in Table 1, the vast majority of controlled studies published to date regarding the effects of yoga for DM2 have been conducted in India, underscoring the need for rigorous trials in other racial/ethnic groups and in other developed and developing countries. Finally, as discussed in more detail below and reflected in the study quality scores (Table 2), the methodological limitations characterizing existing studies highlight the need for additional high-quality RCTs that address these concerns.

## 5. Yoga and DM2: Possible Underlying Mechanisms 

**Figure 1:**
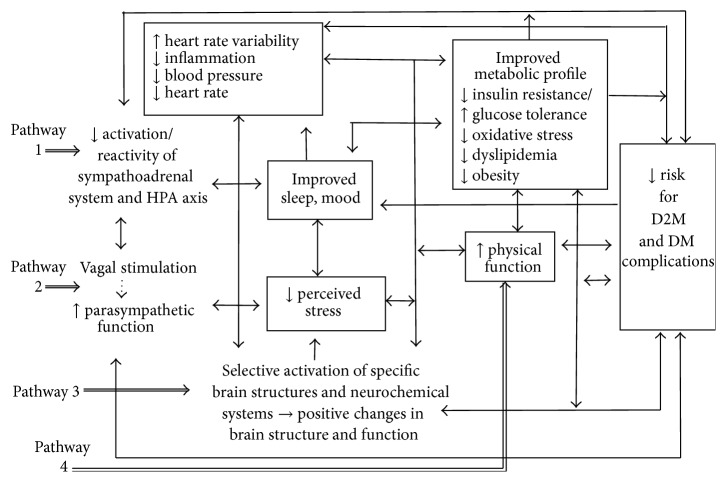
Some pathways by which yoga practices may influence outcomes in those with and at risk for type 2 diabetes (DM2). Figure adapted with permission from Hansen and Innes [132].

Although the mechanisms underlying the apparent beneficial effects of yoga therapy on diabetes risk profiles are not yet well understood, mechanistic pathways are likely complex and interacting. The observed changes may occur through at least four pathways, as depicted in Figure 1 [10, 76, 113, 117, 125, 132].

First, yoga may lessen the negative impact of stress and promote multiple positive downstream effects on metabolic function, neuroendocrine status, and related inflammatory responses and, ultimately, reduce risk for CVD and other vascular complications, by enhancing well-being and reducing reactivity and activation of the HPA axis and the sympathoadrenal system [10, 76, 113, 132]. For instance, recent controlled trials suggest even short term yoga training programs can reduce perceived stress, improve mood, and lower catecholamine and cortisol levels, cardiovascular response to stress, blood pressure, and other indices of sympathetic activation in both healthy and clinical populations, including adults with diabetes [10, 35, 80, 113, 132–134]. Sympathetic activation, HPA dysregulation, and psychological distress have also been linked to the development and exacerbation of several risk factors for DM2 [10, 76, 80, 83, 88, 132, 133, 135, 136], including insulin resistance, impaired glucose tolerance, hypertension, dyslipidemia, central obesity, and proinflammatory and prothrombotic states. These conditions have been shown to have adverse effects on metabolic control and neuroendocrine function and have also been implicated in elevated risk for DM2, CVD, and other vascular disorders [10, 76, 80, 83, 88, 132, 133, 135].

Second, yogic practices may shift the autonomic nervous system balance from primarily sympathetic to parasympathetic, by directly enhancing parasympathetic output, possibly via vagal stimulation [76, 122, 137], resulting in positive changes in cardiovagal function and associated neuroendocrine, hemodynamic, and inflammatory profiles, in sleep and affect, and in related downstream metabolic parameters (Figure 1, pathway 2) [10, 80, 113, 133]. For example, recent controlled studies in adults with DM2, CVD, hypertension, and other chronic conditions, as well as in healthy populations, have shown yogic exercises to reduce resting heart rate, enhance baroreflex sensitivity, and increase heart rate variability, both immediately and following short term (6–12 weeks) yoga programs [10, 113, 133, 138, 139]. Heart rate variability, resting heart rate, and baroreflex sensitivity are widely used markers of cardiovagal autonomic function, parasympathetic activation, and autonomic balance [76, 120, 140, 141] and are thought to in part reflect stimulation of the vagus nerve [76, 122, 137]. These factors have been strongly linked to increased risk for DM2, as well as CVD [10, 76, 80, 142].

Third, yoga may also promote favorable changes in autonomic balance, memory and mood, neurological structure and function, and related metabolic and inflammatory responses by selectively activating specific brain structures and neurochemical systems related to attention and positive affect (Figure 1, pathway 3), as suggested by recent neurophysiological and neuroimaging research findings [143–146]. And finally, yogic practices may improve both metabolic and psychological risk profiles, support increased physical activity, enhance neuroendocrine function, improve body composition, and promote weight loss by increasing strength, overall fitness, and physical function (Figure 1, pathway 4). Yoga may also reduce CVD risk in other ways. For instance, by reducing stress and leading to improved sleep and mood, yoga may indirectly improve CVD risk profiles by leading to healthier lifestyle choices and enhanced self-care [147]. Yoga may also increase resilience to stress, a factor that has been linked to improved outcomes in DM2 [148, 149], although study findings to date have been inconsistent [150–152].

In addition, yoga may benefit those with DM2 indirectly by encouraging improvements in health-related attitudes and lifestyle choices [147, 153, 154] and by providing a source of social support, a factor linked to improved diabetes self-care and clinical outcomes [155, 156]. As suggested by recent studies of patients with heart failure, chronic obstructive pulmonary disease, and DM1 [157–159], yogic breathing practices may also, by increasing arterial and tissue oxygenation, alleviate underlying hypoxia and thereby enhance autonomic cardiac and respiratory function and related endpoints in adults with diabetes [157–160]. Finally, several recent genomic investigations in dementia caregivers [161, 162] and healthy adults [163, 164] suggest that yogic meditative practices can slow cellular aging and induce beneficial epigenetic changes in pathways regulating inflammation, oxidative stress, energy metabolism, insulin secretion, mitochondrial function, and other related factors; these changes may, in turn, help buffer the deleterious effects of stress, improve glucose control, enhance mood, sleep, and autonomic function, reduce blood pressure, and promote improvements in other related risk factors of relevance to DM2 management [76, 165, 166].

## 6. Study Limitations and Directions for Future Research

This systematic review was limited in that it did not include unpublished studies, dissertations, or abstracts. In addition, some controlled trials of yogic practices may have been missed if the article did not make reference to the word yoga or yogic, although every effort was made to locate all relevant studies meeting our inclusion criteria. Many of the studies reviewed here suffer from methodological problems, poor reporting, and other limitations that render interpretation of findings challenging and the formation of definitive conclusions difficult. PEDro scores ranged from a low of 1 [38, 40, 43, 64] to a high of 6 [57, 59, 61, 62, 66, 67] points, out of 10 (Table 2). Importantly, participants were not randomly assigned to treatment in more than half of the studies, increasing risk for selection bias, and concealment of treatment allocation was reported in only 3 [57, 61, 65] of the 25 trials. Among the 12 RCTs, only 2 [57, 65] specified how randomization was performed, and only 7 [52–56, 58, 59, 61–63, 66–68, 70] demonstrated baseline comparability between the groups regarding the most important prognostic indicators. Interestingly, 8 [39, 44–50] of the 13 NRCTs also exhibited groups that were similar at baseline.

Given the nature of the intervention, blinding of the therapists and participants was likely not feasible; understandably, none of the studies satisfied these two PEDro criteria. This deficiency in blinding potential places even greater importance on ensuring that outcome assessors are blinded to group assignment. Unfortunately, blinded outcome assessment was specifically addressed in only two trials [57, 66, 67], raising the possibility of information bias. Retention was not always reported and varied widely (from 60% to 100%), with less than half of the studies (*n* = 11 [39, 42, 45, 50, 56, 58–62, 66, 67, 69, 70]) reporting outcome data from 85% or more of those initially assigned to a group. Reasons for dropout were rarely specified. Only five studies included specific data on participant adherence [45, 56, 65, 67, 70], and even in these studies, adherence was highly variable, ranging from excellent [45, 67] to very poor [65]; moreover, for one study [53], adherence data were reported for only a subset of participants [56].

In some articles, details regarding analytic methods were sparse, and/or analyses were inadequate. For example, while all studies in this review included a comparator group, between group comparisons were not presented in 10 of the 25 studies, including 2 [60, 64] RCTs. In those studies documenting attrition, only eight [39, 42, 50, 57, 59, 60, 65, 69] indicated all participants completed the study as designed or that intention to treat analyses had been conducted. Additionally, although all but two [64, 65] studies provided point measures and measures of variability, other data were often incompletely or confusingly presented. Furthermore, in many papers, the study population, participant recruitment, yoga-based intervention, and/or comparator condition were poorly described, rendering replication of these studies challenging and limiting conclusions. Most of the trials were relatively small, with 56% of them including 60 or fewer participants and 24% including 40 or less (Table 1), although, overall, sample size has been increasing in the last decade, especially the last 5 years.

While the investigations published to date have yielded overall positive results, the considerable heterogeneity in study design, intervention, comparator condition, and duration characterizing the 25 trials render direct comparisons across studies challenging. Participant characteristics also varied substantially, both within and between studies. Nonetheless, while this heterogeneity precludes specific recommendations for particular yoga programs or subpopulations, the consistently positive findings reported in multiple and varied samples of adults with DM2 suggest that yoga may be helpful for a broad range of patient groups. Of the 25 eligible controlled studies identified in this review, only five were conducted in countries other than India, including two in the UK, two in Cuba, and one in Iran. Rigorous trials in Western and many other countries, including many developing nations, where DM2 is now reaching epidemic proportions, remain few. It thus remains unclear if the positive findings reported in existing studies can be generalized to other populations, cultures, and/or regions. As indicated earlier, the mechanisms underlying the observed benefits of yoga are still poorly understood. Finally, rigorous dosing, cost-effectiveness, and long-term follow-up studies are lacking, as are trials assessing the effects of yoga on clinical endpoints such as diabetes-related morbidity and mortality. All of these areas warrant future research.

## 7. Conclusion

In conclusion, the findings of controlled trials published to date suggest that yogic practices may promote significant improvements in several indices of major importance in the management of DM2, including glycemic control, lipid levels, and body composition. More limited data suggest that yoga may also lower oxidative stress and blood pressure, enhance pulmonary and nervous system function, improve mood, sleep, and quality of life, and reduce medication use in adults with DM2. However, given the methodological limitations and heterogeneity of existing studies, findings must be interpreted with caution. Additional high-quality investigations are required to confirm and further elucidate the potential therapeutic benefits of standardized yoga programs in populations with DM2 [10, 132].

## Figures and Tables

**Table 1:** Characteristics of eligible studies investigating the effects of yoga-based programs in adults with diabetes (25 controlled trials, including 12 randomized (RCTs) and 13 nonrandomized (NRCTs)).

	NRCTs (*N*)	RCTs (*N*)	Total	
			*N*	%
Participant characteristics				
Target population: adults with				
Type 2 diabetes only	12	12	24	96.0%
Unspecified diabetes	1	0	1	4.0%
One gender only specified	0	2	2	8.0%
Excluding those on DM meds				
Yes	2	0	2	8.0%
Not specified	0	1	1	4.0%
Excluding those with DM complications				
Yes	9	10	19	76.0%
No	1	1	2	8.0%
Not specified	3	1	4	16.0%
Age range in years				
≥18–26	0	3	3	12.0%
30/35–55/60/65	6	2	8	32.0%
40/45–55/60	3	2	5	20.0%
40/45–65/70/75	3	2	5	20.0%
50–70/>60 y	0	2	2	8.0%
Not specified	1	1	2	8.0%
Years since DM diagnosis				
≥0-1 year	0	2	2	8.0%
1/2 years	1	0	1	4.0%
2–5 years	0	1	1	4.0%
5–10 years	0	1	1	4.0%
0/1–10 years	7	1	8	32.0%
>15 years	0	2	2	8.0%
Not specified	5	5	10	40.0%
Sample size				
<25	0	2	2	8.0%
25–40	2	2	4	16.0%
41–60	4	4	8	32.0%
>60	7	4	11	44.0%
Location				
India	13	7	20	80.0%
UK	0	2	2	8.0%
Cuba	0	2	2	8.0%
Iran	0	1	1	4.0%
Year published				
2010–2014	7	7	14	56.0%
2005–2009	2	3	5	20.0%
2000–2004	3	1	4	16.0%
Prior to 2000	1	1	2	8.0%
Yoga intervention^*∗*^				
Yoga-based program alone				
Including asanas	11	10	21	84.0%
Not including asanas	0	2	2	8.0%
Yoga combined with other interventions				
Including asanas	3	0	3	12.0%
Not including asanas	0	0	0	0.0%
Duration				
<8 weeks	6	1	7	28.0%
12 weeks/3 months	5	7	12	48.0%
4–6 months	2	2	4	16.0%
>6 months	0	2	2	8.0%
Frequency of practice^*¥*^				
<3x/week	0	1	1	4.3%
3x/week	1	3	4	17.4%
4-5x/week	2	1	3	13.0%
6-7x/week	8	7	15	65.2%
Program structure^*¥¥*^				
Classes only	9	7	16	69.6%
Classes combined with home practice	1	5	6	26.1%
Training session combined with home practice	1	0	1	4.3%
Comparison condition^*∗∗*^				
Usual care/no treatment	11	8	19	76.0%
Attention control	0	1	1	4.0%
Active comparator	3	5	8	32.0%
>1 control	1	2	3	12.0%

^*∗*^Including two yoga-based interventions tested within the same study [38].

^*¥*^Practice frequency not specified in 2 NRCTs [39, 40].

^*¥¥*^Information on program structure lacking in two NRCT's [39, 41].

^*∗∗*^Numbers add up to more than 25, as 3 studies included more than one comparator.

**Table 2:** Summary of study characteristics and major findings.

First author, year, and location	Tx duration	Study population	*N* enrolled/completed (per group) [% retention]	Yoga intervention	Comparison condition	Assessment times	Major findings	PEDro scale score (range 0–10)
Nonrandomized controlled trials (NRCTs)								

Dash, 2014, India [42]	40 days	Adults 40–60 yo w/DM2 for 0–10 y; Excl: BMI > 25; DM1; DM2 w/nephropathy, CAD, retinopathy, alcoholism; practicing any yoga; 37% F	60 (30Y, 30C) [100%]^#^	Yoga (AS, PR, SH, and M): 30–40 min/d; + prescribed diet, oral meds	Prescribed diet, oral meds	Before and after: FBG, PPBG, A1c, TC, Tg, LDL, HDL, and VLDL	**Yoga:** Sig ↓ in FBG, PPBG, A1c, TC, Tg, LDL, VLDL, Sig ↑ HDL; **C:** Sig ↓ in FBG, PPBG, VLDL; **Y vs. C**: Sig ↓ in FBG, PPBG, A1c, TC. TG, LDL, Sig ↑ HDL	4

Popli, 2014, India [43]	6 mos	Adults 30–60 yo w/DM2, no previous yoga experience; Excl: Pts already on oral hypoglycemic meds or insulin, w/alcoholism, BG > 300 mg/dL, or end-organ damage (e.g., retinal detachment, nephropathy, and peripheral neuropathy)	130 (80Y, 50C)	Yoga (AS, PR): 1 hr with yoga trainer, 5 d/wk for 1st month, and then continuing practice at home for remaining 5 mos	No treatment	Monthly: FBG; 3x/mo: PPBG, A1c	**Yoga:** Sig ↓ in FBG, PPBG, A1c; **C:** NS	1

Bindra, 2013, India [39]	up to 90 days	Adults 35–65 yo w/DM2 for not >10 y and no complications; Excl: those w/RA, cancer, TB, and MI and not willing to do yoga	100 (50Y, 50C) [100%]^#^	Yoga (not described) + oral DM meds	Oral DM meds only	Before and after: FBG, A1c, TC, LDL, HDL, and Tg	**Baseline yoga versus C:** NS differences between the groups; **After 90 days, Yoga versus C**: Signif. differences between the groups in FBG, A1c, TC, LDL, HDL	5

Balaji, 2011, India [44]	3 mos	Uncomplicated DM2 Pts 40–55 yo w/DM duration 1–10 y	44 (22Y, 22C) [100%]^#^	Yoga (AS, PR, SH): daily for 1 hr; subgroup T1 (*n* = 16): + oral drugs; subgroup T2 (*n* = 6): + oral drugs and insulin	22 controls	Before and after: FBG, PPBG, A1c, TC, Tg, LDL, HDL, and insulin requirement/day (subgroup T2), wt, BMI, W : H	**Yoga T1:** Sig ↓ in FBG, A1c, Tg, LDL, wt, BMI, W : H; **Yoga T2**: Sig ↓ in FBG, PPBG, A1c, Tg, LDL, wt, BMI, W : H, insulin/d; **C:** NS	2

Hegde, 2011, India [45]	3 mos	DM clinic Pts w/DM2; age: 40–75 y; Excl: smoking, alcoholism	123/120 (60Y, 63C); strat'd by complications [98%]^*∗∗∗*^	Yoga (AS, PR, and SH): at least 3 d/wk; + standard care	Standard care (given general oral and written information about diet and exercise)	Before and after: FBG, PPBG, A1c, oxidative stress (MDA, glutathione, SOD, Vit. C, and Vit. E), BMI, waist circumference, W : H, and BP	**Y versus C**: Sig ↓ in FBG, PPBG, A1c, BMI, MDA, Sig ↑ glutathione, Vit. C	4

Kyizom, 2010, India [46]	45 days; groups matched for age and sex	DM clinic Pts, 35–60 yo, w/uncomplicated DM2 from 2–10 y	60 (30Y, 30C) [100%]^#^	Yoga (AS, PR, and SH): daily for 45 min/d; + standard care	Standard care	Before and after: FBG, PPBG, event-related evoked potentials at frontal (Fz), vertex (Cz), and parietal (Pz) areas, peak latencies, and baseline to peak amplitudes of N2 and P3	**Yoga:** Sig ↓ in FBG, PPBG; Sig ↓ in latency of N2 at Cz and P300 at all the montages (Fz, Cz, and Pz); Sig improvement in amplitude of wave N2 at Cz and P300 at Pz montage **C:** NS	2

Malhotra, 2010, India [47]	40 days; controls matched on age and DM severity	Pts from endocrine metabolic clinic w/DM2 for 0–10 y, 30–60 yo, on recommended diet and oral DM meds; Excl: CAD, nephropathy, and proliferative retinopathy	106 (56Y, 50C)/64 (26Y, 38C) [60%]	Yoga (AS, PR, and SH): 40–60 min/d; + diet and DM meds	Standard care: meds, diet, and light exercises (walking)	Before and after: FBG, PPBG, TC, Tg, HDL, LDL, VLDL, MDA, and insulin; Wt, W : H, BMI, LBM, and BSA; SBP, DBP, HR, and QT interval; SVC, FEV1, FVC, FEV1 : FVC ratio, PEFR, and MVV; median nerve conduction velocity (m/sec), amplitude (mV), and latency (msec) measured proximally (at elbow) and distally (at wrist)	**Yoga:** Sig ↓ in FBG, TC, Tg, MDA, SBP, DBP, heart rate, W : H; Sig improvement in pulmonary, CV fxn, Sig ↑ in left hand nerve conduction velocity and amplitude (distal) **C:** Sig ↓ in LBM (worsening), nerve conduction amplitude (right proximal), Sig ↑ in Tg (worsening), insulin (in 4 pts), HR, PEFR; **Y versus C**: not analyzed	2

Mahapure, 2008, India [41]	6 wks	Pts 40–58 yo, w/DM2 on regular diet and antidiabetic drug regimen	40 (30Y, 10C) [100%]^#^	Yoga (AS, PR, SH); 1 hr/d, every d except Sundays; + standard care	Standard care: diet and meds	Before and after: FBG, A1c, and SOD	**Yoga:** Sig ↓ in FBG, A1c, Sig ↑ SOD; **C**: NS; **Y versus C**: Sig ↓ in FBG, A1c, Sig ↑ SOD	3

Singh, 2008, India [48]	45 days	Pts 35–60 yo, w/uncomplicated DM2 for 1–10 y	60 (30Y, 30C) [100%]^#^	Yoga (AS, PR, and SH): daily for 45 min/d; + standard care	Standard care (conventional meds)	Before and after: FBG, PPBG, A1c, TC, Tg, LDL, VLDL, HDL, insulin, wt, and BMI	**Yoga:** Sig ↓ in Wt, FBG, PPBG, insulin, TC, Tg, VLDL, LDL; Sig ↑ in HDL; **C**: Sig ↑ Wt; **Y versus C**: not given	2

Agte, 2004, India [40]	4 mos	Previously diagnosed DM2 Pts 45–65 yo; having stabilized glucose levels, and taking conventional prescribed medications; BMI: 25.4 (Y), 25.6 (C); 53% F	87 (57Y, 30C)/65 (35Y, 30C) [75%]	SKY + PR, AS, and M; interactive discussions on stress-free living; nutritional counseling w/emphasis on eating fresh fruits and vegetables; 6 d course	Without SKY training	Before and after: FBG, PPBG, A1c, TC, Tg, HDL, and MDA	**Yoga**: Sig ↓ in FBG, PPBG, TC, Tg, MDA; **C:** NS; **Y versus C**: not presented	1

Agrawal, 2003, India [49]	3 mos	Pts randomly selected from diabetes clinic (type of DM not specified, but background implies that study targeted DM2); Excl: Pts w/liver disease, alcoholism, malnutrition, thyrotoxicosis, TB, or who were noncooperative	200/154 (82Y, 72C) [77%]	Yogic lifestyle program: health rejuvenation exercises (5 min), AS (18 min), abdominal exercises (7 min), and R/M/PR (30 min); at least 5 d/wk + diet	Standard medical tx or standard home exercise program	Before and after: FBG, A1c, TC, HDL, LDL, VLDL, BP, BMI, W : H, QOL (satisfaction, impact, and worry), psychological assessment, self-evaluation, doses of metformin, glipizide, and insulin, BP, and renal function	**Y versus C**: Sig ↓ FBG, A1c, BMI, W : H, TC, LDL, VLDL, SBP, DBP; metformin, glipizide, and insulin doses; QOL satisfaction, impact, and worry scores, psychological assessment, and self-evaluation; Sig ↑ HDL	3

Malhotra, 2002, India [50]	40 days; controls matched on age, sex, SES, and DM severity	DM2 for 0–10 y; 30–60 yo; Excl: cardiac, renal, and proliferative retinal complications	40 (20Y, 20C) [100%]	Supervised yoga (AS, PR, and SH): daily for 30–40 min/d; + medication, diet	Medication, diet, and light exercise (walking)	Before and after 40-day treatment: *median nerve* conduction velocity (m/sec), amplitude (mV), and latency (msec) measured proximally (at elbow) and distally (at wrist)	**Yoga**: Sig ↑ conduction velocity in left hand; **C**: Sig ↓ in amplitude at right elbow (proximal)	4

Khare, 1999, India [38]	3 mos, non-DM controls matched for age, sex	60 DM2 Pts (DM for 1-2 y) from DM clinics (in yoga/diet groups), 20 non-DM matched on age, sex; 40–70 y on vegetarian diet	80 (20, 20, 20, 20) [100%]^#^	(1) Yoga: AS (3 poses, 2–5 min each) and SH (20 min) only (2) Yoga + strict diet Yoga: 2x/d	(1) No intervention (non-DM adults) (2) Strict diet alone (20 DM adults)	Baseline and 1, 2, and 3 months: BG, serum fructosamine, TC, and Wt	**Yoga:** after 3 mos, sig ↓ BG, Fruc, TC, and Wt **Yoga + diet**: after 3 mos, sig ↓ BG, Fruc, TC, and Wt **Diet**: after 3 mos, sig ↓ TC and Wt **non-DM, no tx**: after 3 mos, NS	1

Randomized controlled trials (RCTs)								

Habibi, 2013, Iran [51, 52]	12 wks	Women w/DM2, 45–60 yo, not taking insulin; without DM complications or hx of CVD	26 (16Y, 10C) [100%]^#^	Yoga: 75 min, 3 d/wk	Standard care	Before and after: BP, FBG, F insulin, TC, HDL, LDL, leptin, Wt, and BMI	**Y versus C**: Sig ↓ in FBG, insulin, Tg	4^†^ [52]

Jyotsna, 2012, India [53–56]	6 mos	Adults w/DM2 plus: lifestyle modification, A1c of 6–9%, and oral hypoglycemic agents for past 6 months; Excl: Pts w/retinopathy, glaucoma, uncontrolled HTN, CAD, overt complications of diabetes, and nephropathy; mean age (y): 49.92 ± 11.46 (yoga), 47.25 ± 10.80 (control)	120 (64Y, 56C) [100%]^#^	Sudarshan Kriya yoga (SKY: a rhythmic cyclical breathing, preceded by PR); 3 d group training program followed by classes 1x/wk (long kriya) and daily home practice (short kriya) + standard care	Standard care (oral antidiabetic drugs and diet and exercise advice)	Before and 6 mos after tx: CAFTs, FBG, PPBG, and A1c; Before and 3 and 6 mos after tx: QOL (domains: (1) physical health, (2) psychological well-being, (3) social relationships, and (4) environment)	**Yoga:** Sig ↑ Symp fxn; **C:** NS; **Y versus C:** Sig ↑ overall cardiac autonomic fxn; Sig ↓ PPBG; Sig ↑ QOL: 3 mos after: domains 1 and 2 and total QOL; 6 mos after: domains 2 and 4 and total QOL	4^†^ [53, 54]; 4 [55]; 5^†^ [56]

Nagarathna, 2012, India [57]	9 mos	Pts >25 yo with DM2 >1 y (FBG >120 mg% when dx), stable dose of oral hypoglycemic agents or insulin for at least 3 wks, no prior yoga practice, no major complications; mean age: 52.4 y; 31% F	277 (141Y, 136C)/173 (88Y, 85C) [62%]	Yoga (AS, PR, M, devotional sessions, and lectures): 1 hr/d, 5 d/wk for 12 weeks [cleansing techniques (kriyas) performed 1x/wk]; one 2 hr class/wk and 1 hr daily home practice for 9 mos	Exercises and walking designed to achieve a comparable intensity of physical exertion, nonyogic breathing exercises, supine rest, and lectures: 1 hr/d, 5 d/wk for 12 wks; one 2-hour class/wk and 1-hour daily home practice for 9 mos	Before and after: TC, Tg, HDL, LDL, VLDL, FBG, PPBG, A1c, and oral hypoglycemic medication requirement	**Yoga:** Sig ↓ meds, FBG, PPBG, A1c, Tg, TC, LDL, VLDL; Sig ↑ HDL; **C:** Sig ↓ PPBG, A1c, Tg, TC, VLDL; **Y versus C**: Sig ↓ meds, LDL; Sig ↑ HDL	6

Shantakumari, 2012, India [58, 59]	3 mos	Outputs with DM2 + HTN and dyslipidemia, 35–55 yo; Excl those w/known retinopathy, nephropathy, CAD, and cerebrovascular diseases; avg DM duration: 5–10 y; 48% F	100 (50Y, 50C) [100%]^#^	Yoga: 1 hr daily; AS (30–35 min), PR (10 min), and M (15 min); + standard care	Standard care (oral hypoglycemic drugs, no yoga)	Before and after: FBG, PPBG, TC, HDL, LDL, Tg, Wt, W : H, BMI, SBP, and DBP	**Yoga:** Sig ↓ Wt, W : H, Tg, TC, LDL; **C:** Sig ↑ Wt	6^†^ [59]; 5^†^ [58]

Subramaniyan, 2012, India [60]	15 days	Males ≥18 yo w/DM2 on standard care and medically eligible for walking/yoga per physician; age: 55% were 31–40 yo	20 [100%]^#^	Yoga: 60 min (6-7 a.m.) daily; AS (~30 min), sun salutation (~6 min), SH (~25 min) + routine meds	Brisk walking: 60 min (6-7 a.m.) daily + routine meds	Before and after: FBG	**Yoga:** Sig ↓ in FBG **Walking:** Sig ↓ in FBG	4

Vaishali, 2011, India [61, 62]	12 wks	Diabetic clinic Pts, >60 yo, w/DM2 >15 y and ≥1 metabolic risk factor (high FBG, pre-HTN, overwt/obese, high TC), on antidiabetic meds >10 y; Excl: uncontrolled HTN, severe neuropathy, hx of foot lesions, unstable proliferative retinopathy, and nephropathy	60/57 [95%]	Yoga (individualized AS, PR, and SH): 45–60 min under supervision, 6 d/wk	Educational group (general healthy lifestyle and exercise): 1x/mo	Before and after: FBG, A1c, TC, Tg, LDL, and HDL	**Yoga: Sig ↓ in FBG, A1c, TC, Tg, LDL; Sig ↑ HDL** **C: NS** **Y versus C**: Sig ↓ in A1c, FBG, TC, Tg, LDL; Sig ↑ HDL	6^†^ [61]; 6 [62]

Pardasany, 2010, India [63]	12 wks	Adults w/DM2, 40–60 yo; Excl: hx of renal disease, arthritis, HBP, intermittent claudication, foot injury or ulcers, breathlessness, and cardiac disease; 38% F	45 (15, 15, 15) [100%]^#^	Hatha yoga (12 AS and 6/7 PR): 3x/wk; oral hypoglycemic meds	(1) Yang-style tai chi (TC, 24 forms): 3x/wk, oral hypoglycemic meds; (2) Oral hypoglycemic meds, no exercise (C)	Before and after: FBG, PPBG, A1c, TC, and LDL	**Yoga:** Sig ↓ FBG, PPBG, A1c, TC, LDL **TC:** Sig ↓ FBG, PPBG, A1c, TC, LDL **C:** NS **Y versus C: **Sig ↓ FBG, PPBG, TC, LDL; **TC versus C**: Sig ↓ FBG, PPBG, A1c, TC; **Tai chi versus Y**: NS	4^†^

Amita, 2009, India [64]	Up to 90 days	Middle aged (35–65 yo), DM2 Pts on oral hypoglycemics; Excl: >200 mg/dL FBG, >300 mg/dL PPBG; hx of DM complications or other systemic conditions; 29% F	41 (20Y, 21C) [100%]^#^	Yoga nidra (deep relaxation): 45 min, daily	Standard care (no yoga)	Every 30 days: FBG, PPBG, and symptoms related to DM (i.e., insomnia, palpitations, sweating, distress, headache, and anxiety)	**Yoga:** Sig ↓ FBG (60 and 90 d), PPBG (90 d), total symptoms (insomnia, distress, etc. after 3 mos); **C:** not given	1^†^

Skoro-Kondza, 2009, UK (London) [65]	12 wks	Adults (>18 yo) w/DM2, not on insulin; most well-controlled (A1c *x* = 6.9); mean duration of DM2 (y): 30 ± 5; mean age (y): 60 ± 10; nonwhite: 55% at one site, 40% at the other site; 61% F	59 [100%]^#*∗*^	Yoga (PR, AS, and SH): 90 min class, 3x/wk	Wait list (both groups given leaflets on healthy lifestyle and encouraged to exercise)	Before and after and six months later: A1c, wt, waist circumference, W : H, BMI, TC, LDL, Tg, HDL, SBP, and DBP (used to calculate cardiovascular risk score); diabetes-related QOL, and self-efficacy.	**Yoga:** NS; **C:** NS: **Y versus C**: NS **Note**:Quantitative data provided only for A1c	4^†^

Gordon, 2008, Cuba [66–68]	24 wks, age and sex matched controls	Pts 40–70 yo w/DM2 from 1–10 y, w/at least 3 mos of training in DM education, exercise, diet, and medication according to IDF recommendations; no severe complications, nonsmoker, nonalcoholic	231 (77 Pts: 62 females, 15 males per group) [100%]^#*∗∗∗*^	Yoga: PR (20 min), warm-up exercises (25 min), AS (60 min), and SH (15 min); 1 class/wk for 24 wks plus home yoga exercise (followed IDF criteria, with yoga as exercise)	(1) PT: warm-up exercises (15 min), aerobic walking (30 min), flexibility exercises (20 min), aerobic dance (20 min), games (25 min), warm-down (10 min); 1 class/wk for 24 wks plus home exercise 3-4x/wk (followed IDF criteria); (2) Control: tx plan as per their doctors, no active exercise tx	Baseline and 3 and 6 mos: FBG, A1c, insulin, TC, Tg, HDL, LDL, VLDL, BMI, MDA, PLA2 activity, POX, and SOD and catalase activity; microalbuminuria, creatinine; cortisol, TSH, T3, and T4	**Yoga:** 3 mos: Sig ↓ FBG, TC; 6 mos: Sig ↓ FBG, A1c, TC, VLDL, microalbuminuria, MDA; Sig ↑ SOD; **PT:** 3 mos: Sig ↓ FBG, TC; 6 mos: Sig ↓ FBG, TC, VLDL, microalbuminuria, MDA; Sig ↑ SOD; **C:** 3 mos: NS; 6 mos: NS; **Y versus C**: 3 mos: Sig ↓ FBG, A1c, TC; 6 mos: Sig ↓ FBG, A1c, TC, MDA, BMI, microalbuminuria, Sig ↑ % insulin receptor binding **PT versus C**: 3 mos: Sig ↓ FBG, A1c, TC; 6 mos: Sig ↓ FBG, A1c, TC, MDA, BMI, microalbuminuria, Sig ↑ % insulin receptor binding	6^†^ [66, 67]; 4^†^ [68]

Céspedes, 2002, Cuba [69]	12 mos	DM2 patients, 50–70 y, DM duration 2–5 y, without malnutrition or severe complications, in good mental health	40 (22 Y, 18 C) [100%]^#^	Moderate intensity yoga (PR, AS): 60 min class, 3x/wk + lifestyle advice, soybean-rich diet	Moderate intensity aerobic exercise: 60 min class, 3x/wk + lifestyle advice, soybean-rich diet	Before and after: BG, TC, HDL, LDL, Tg, and creatinine	**Yoga:** Sig ↓ TC, LDL, Tg, creatinine; Sig ↑ HDL; **PT: **Sig ↓ TC, LDL, Tg, creatinine; Sig ↑ HDL; **Y versus C:** Sig ↓ TC, LDL, Tg, creatinine; Sig ↑ HDL	5

Monro, 1992, UK [70]	12 wks	DM2 controlled with meds (*N* = 13; yoga group = 8) or diet (*N* = 8); Excl: end stage liver or kidney disease or congestive cardiac failure; age (y): 45–67	21 (11Y, 10C) [100%]^*∗∗*^	Yoga (PR, AS, and R) + standard care: 90 min, classes offered 5x/wk (most attended classes 1-2x/wk and practiced at home 1-2x/wk)	Standard care (continuing on medication, diet)	Before and after: FBG and A1c	**Y versus C**: Sig ↓ in A1c, FBG	5^†^

^#^Assumed (retention not specifically reported), ^*∗*^adherence very low, ^*∗∗*^adherence moderate, ^*∗∗∗*^adherence excellent, and ^†^score from PEDro database.

A1c: glycosylated hemoglobin A1c, AHA: American Heart Association, AS: yoga asanas or postures, avg: average, BF: biofeedback, BP: blood pressure, BSA: body surface area, C: control, CAD: coronary artery disease, CAFT: cardiac autonomic function tests, cal: calorie, Clin: clinical, comp: composition, CVD: cardiovascular disease, d: day, DM: diabetes mellitus, Excl: excluded, F: female, FBG: fasting blood glucose, FEV1: forced expiratory value in first second, FVC: forced vital capacity, h: hour, HDL: high density lipoprotein, HR: heart rate, HTN: hypertension, hx: history, IFN*γ*: interferon gamma, a cytokine critical for innate and adaptive immunity against viral and intracellular bacterial infections and for tumor control, IR: insulin resistance (markers of), KR: kriyas or cleansing exercises, LBM: lean body mass, LDL: low density lipoprotein, M: meditation, MDA: malondialdehyde, MI: myocardial infarction, mo: month, MVV: maximal voluntary ventilation, PEFR: peak expiratory flow rate, PLA2: phospholipase A2, PMR: progressive muscle relaxation, POX: protein oxidation, PPBG: postprandial blood glucose, PR: pranayama or yogic breathing exercises, QOL: quality of life, R: relaxation poses (nonspecified), RA: rheumatoid arthritis, Resid: residential, SH: shavasana or corpse pose, a traditional yoga relaxation pose, Sig: significant, SNS/PNS: markers of sympathetic/parasympathetic activation, including heart rate and catecholamine levels, SOD: superoxide dismutase, SVC: slow vital capacity, TB: tuberculosis, TC: total cholesterol, Tg: triglycerides, veg: vegetarian, VLDL: very low density lipoprotein, W : H: waist-hip ratio, wk: week, wt: weight, y: years, and yo: years old.

**Table 3:** Observed percent change with yoga in metabolic indices, body weight, and blood pressure among adults with type 2 diabetes (*N* = 23 controlled trials), stratified by study design and analytic comparisons presented (before/after, intergroup). Only studies reporting significant changes included.

Findings, by clinical measure	Study design			
	Nonrandomized controlled trials		Randomized controlled trials	
	Yoga versus baseline	Yoga versus controls	Yoga versus baseline	Yoga versus controls
*Measures of insulin resistance*				
Fasting glucose	14.33–33.23% [38, 40–44, 46–48]	9.87–29.75% [39, 41, 42, 45, 49]	7.21–27.53% [57, 60, 63, 64, 66]	13.46–36.2% [51, 61, 63, 66, 70]
Postprandial glucose	5.92–38.65% [40, 42–44, 46, 48]	11.61–35.5% [42, 45]	7.03–18.89% [57, 63, 64]	11.17–14.22% [55, 63]
Fasting glycosylated hemoglobin (HbA1c)	1.55–21.23% [41–44]	1.03–7.44% [39, 41, 42, 45, 49]	0.31–14.17% [57, 63, 67]	7.77–17.78% [61, 67, 70]
*Blood lipid profiles*				
Total cholesterol	5.97–14.61% [38, 40, 42, 47, 48]	4.11–8.63% [39, 42, 49]	1.37–37.01% [57, 58, 63, 66, 69]	4.74–18.3% [61, 63, 66, 69]
Triglycerides	3.97–16.35% [40, 42, 44, 47, 48]	3.53% [42]	14.33–38.0% [57, 58, 69]	1.06–10.96% [51, 61, 69]
Low-density lipoprotein (LDL)	2.18–14.88% [42, 44, 48]	1.38–15.05% [39, 42, 49]	8.33–48.45% [57, 58, 63, 69]	3.39–11.42% [57, 61, 63, 69]
High-density lipoprotein (HDL)	8.28–15.21% [42, 48]	5.01–24.96% [39, 42, 49]	6.99–101.59% [57, 69]	9.1–54.92% [57, 61, 69]
Very low-density lipoprotein (VLDL)	15.17–16.06% [42, 48]	13.28% [49]	7.23–22.66% [57, 66]	
*Anthropometric measures*				
BMI	7.52–10.34% [44]	2.72–3.56% [45, 49]		4.32% [67]
Body weight	3.48–6.95% [38, 44, 48]			4.18% [58]
Waist-hip circumference ratio	5.38–8.99% [44, 47]	3.17% [49]		5.32% [58]
*Blood pressure*				
Systolic blood pressure	11.27% [47]	5.03% [49]		
Diastolic blood pressure	12.92% [47]	3.46% [49]		

**Table 4:** Recent published meta-analyses regarding effects of yoga on risk indices relevant to T2DM, summarized findings.

Measure	First author, year	Population	Included studies			Comparator	Mean difference (95% CI)	*P*
			Design	Number	Total Pts			
*Glucose tolerance*								
Fasting blood glucose (mg/dL)	Cramer, 2014 [34]	DM2	RCTs	7	525	Usual care	−25.56 (−39.60, −11.53)	<0.01
	Chu, 2014 [71]	DM or MS	RCTs	6	315	All	−8.77 (−26.77, 9.23)	0.34
HbA1c	Cramer, 2014 [34]	DM2	RCTs	7	550	Usual care	−0.49 (−1.03, 0.05)	0.07
*Lipid profiles*								
Total cholesterol	Cramer, 2014 [34]	DM2	RCTs	5	516	Usual care	−13.09 (−19.60, −6.59)	<0.01
	Cramer, 2014 [34]	DM2	RCTs	2	317	Exercise	−8.08 (−19.20, 3.03)	0.21
	Chu, 2014 [71]	DM or MS	RCTs	5	365	All	−20.98 (−33.23, −8.72)	0.0008
	Chu, 2014 [71]	CVD risk factors	RCTs	2	109	All	−18.85 (−37.24, −0.46)	0.04
HDL	Cramer, 2014 [34]	DM2	RCTs	5	516	Usual care	5.51 (3.56, 7.47)	<0.01
	Cramer, 2014 [34]	DM2	RCTs	2	317	Exercise	4.24 (1.75, 6.72)	0.01
	Chu, 2014 [71]	DM or MS	RCTs	5	365	All	2.45 (−0.33, 5.24)	0.08
	Chu, 2014 [71]	CVD risk factors	RCTs	2	109	All	3.95 (1.69, 6.21)	0.0006
LDL	Cramer, 2014 [34]	DM2	RCTs	5	515	Usual care	−10.26 (−20.85, 0.32)	0.06
	Cramer, 2014 [34]	DM2	RCTs	2	317	Exercise	−9.24 (−16.55, −1.93)	0.01
	Chu, 2014 [71]	DM or MS	RCTs	5	365	All	−21.27 (−32.93, −9.62)	<0.00001
	Chu, 2014 [71]	CVD risk factors	RCTs	2	109	All	−14.42 (−20.47, −8.36)	0.0003
VLDL	Cramer, 2014 [34]	DM2	RCTs	2	308	Usual care	−4.81 (−6.67, −2.96)	<0.01
Triglycerides	Cramer, 2014 [34]	DM2	RCTs	5	509	Usual care	−23.60 (−36.78, −10.43)	<0.01
	Cramer, 2014 [34]	DM2	RCTs	2	317	Exercise	−22.98 (−60.97, 15.00)	0.24
	Chu, 2014 [71]	DM or MS	RCTs	5	365	All	−18.61 (−32.61, −4.60)	0.009
	Chu, 2014 [71]	CVD risk factors	RCTs	2	109	All	−27.47 (−60.09, 5.15)	0.10
*Body composition*								
Waist-hip ratio	Cramer, 2014 [34]	DM2	RCTs	3	311	Usual care	−0.02 (−0.03, −0.00)	<0.01
BMI	Chu, 2014 [71]	DM or MS	RCTs	3	155	All	−1.63 (−2.25, −1.01)	<0.00001
	Chu, 2014 [71]	CVD risk factors	RCTs	2	77	All	−0.99 (−2.38, 0.40)	0.16
Weight (kg)	Chu, 2014 [71]	DM or MS	RCTs	3	151	All	−3.27 (−4.99, −1.54)	0.0002
	Chu, 2014 [71]	CVD risk factors	RCTs	6	273	All	−1.95 (−5.25, 1.35)	0.25
*Blood pressure (BP) (mmHg)*								
Systolic BP	Cramer, 2014 [34]	DM2	RCTs	3	237	Usual care	−6.87 (−14.68, 0.94)	0.08
	Cramer, 2014 [34]	Non-DM high risk	RCTs	8	347	Usual care	−10.00 (−16.42, −3.59)	<0.01
	Cramer, 2014 [72]	HT	RCTs	6	278	Usual care	−9.65 (−17.66, −2.06)	0.01
	Hagins, 2013 [73]	HT (all)	RCTs, NRCTs	11	431	Usual care	−7.96 (−10.65, −5.27)	0.0002
	Hagins, 2013 [73]	HT (Y incl M, PR, AS)	RCTs, NRCTs	13	656	All	−8.17 (−12.45, −3.89)	NR
	Hagins, 2013 [73]	HT (Y > 58.9 h)	RCTs, NRCTs	6	215	All	−9.73 (−17.66, −1.79)	NR
	Chu, 2014 [71]	DM or MS	RCTs	3	80	All	−9.39 (−15.14, −3.63)	0.001
	Chu, 2014 [71]	CVD risk factors	RCTs	10	461	All	−7.36 (−13.39, −1.33)	0.001
Diastolic BP	Cramer, 2014 [34]	DM2	RCTs	2	210	Usual care	−0.79 (−5.22, 3.65)	0.73
	Cramer, 2014 [34]	Non-DM high risk	RCTs	8	347	Usual care	−7.45 (−12.70, −2.21)	<0.01
	Cramer, 2014 [72]	HT	RCTs	6	278	Usual care	−7.22 (−12.83, −1.62)	0.01
	Hagins, 2013 [73]	HT (all)	RCTs, NRCTs	11	431	Usual care	−6.14 (−9.39, −2.89)	NR
	Hagins, 2013 [73]	HT (Y incl M, PR, AS)	RCTs, NRCTs	13	656	All	−5.52 (−7.92, −3.11)	NR
	Chu, 2014 [71]	DM or MS	RCTs	3	80	All	−5.41 (−8.82, −2.01)	0.002
	Chu, 2014 [71]	CVD risk factors	RCTs	10	461	All	−6.97 (−11.80, −2.13)	0.005
*Autonomic fxn*								
Heart rate	Cramer, 2014 [34]	Non-DM high risk	RCTs	3	133	Usual care	−10.89 (−22.83, 1.04)	0.07

AS: asana, CAD: coronary artery disease, CI: confidence interval, CVD: cardiovascular disease, fxn: function, HT: hypertension, M: meditation, MS: metabolic syndrome, NR: not reported, NRCT: nonrandomized controlled trial, PR: pranayama, Pts: participants, and Y: yoga.
